# Immunohistochemical expression of CD44s in human neuroblastic tumors: Moroccan experience and highlights on current data

**DOI:** 10.1186/1746-1596-8-39

**Published:** 2013-02-27

**Authors:** Imane Tabyaoui, Nadia Tahiri-Jouti, Zineb Serhier, Mohamed Bennani-Othmani, Hicham Sibai, Mohamed Itri, Said Benchekroun, Soumaya Zamiati

**Affiliations:** 1 Laboratory of Genetics and Molecular Pathology, Faculty of Medicine and Pharmacy of Casablanca, Hassan II Aïn Chock University, 19 rue Tarik Ibn Ziad, BP 9154, Casablanca, Morocco; 2 Medical Informatics Laboratory, Faculty of Medicine and Pharmacy of Casablanca, Hassan II Aïn Chock University, Casablanca, Morocco; 3 Department of Pediatric Visceral Surgery, “Harouchi Children’s Hospital”, Ibn Rochd University Medical Center, Casablanca, Morocco; 4 Department of Pediatrics III, “Harouchi Children’s Hospital”, Ibn Rochd University Medical Center, Casablanca, Morocco; 5 Department of Pediatric Oncology and Hematology, “20 Août 1953 Hospital”, Ibn Rochd University Medical Center, Casablanca, Morocco; 6 Department of Pathology, “Ibn Rochd Hospital”, Ibn Rochd University Medical Center, Casablanca, Morocco

**Keywords:** CD44s, Human, Metastasis, Immunohistochemistry, Moroccan, Peripheral neuroblastic tumors, Prognosis, CD44s, Métastases, Immunohistochimie, Marocain, Tumeurs neuroblastiques périphériques, Pronostic

## Abstract

**Background:**

Peripheral neuroblastic tumors (pNTs), including neuroblastoma (NB), ganglioneuroblastoma (GNB) and ganglioneuroma (GN), are extremely heterogeneous pediatric tumors responsible for 15 % of childhood cancer death. The aim of the study was to evaluate the expression of CD44s (‘s’: standard form) cell adhesion molecule by comparison with other specific prognostic markers.

**Methods:**

An immunohistochemical profile of 32 formalin-fixed paraffin-embedded pNTs tissues, diagnosed between January 2007 and December 2010, was carried out.

**Results:**

Our results have demonstrated the association of CD44s negative pNTs cells to lack of differentiation and tumour progression. A significant association between absence of CD44s expression and metastasis in human pNTs has been reported. We also found that expression of CD44s defines subgroups of patients without MYCN amplification as evidenced by its association with low INSS stages, absence of metastasis and favorable Shimada histology.

**Discussion:**

These findings support the thesis of the role of CD44s glycoprotein in the invasive growth potential of neoplastic cells and suggest that its expression could be taken into consideration in the therapeutic approaches targeting metastases.

**Virtual Slides:**

The virtual slide(s) for this article can be found here: http://www.diagnosticpathology.diagnomx.eu/vs/1034403150888863

**Résumé:**

**Introduction:**

les tumeurs neuroblastiques périphériques (TNPs), comprenant le neuroblastome (NB), le ganglioneuroblastome (GNB) et le ganglioneurome (GN), sont des tumeurs pédiatriques extrêmement hétérogènes responsables de 15% des décès par cancer chez les enfants. Le but de cette étude était d’évaluer l’expression de la molécule d’adhésion cellulaire CD44s (‘s’: pour standard) par rapport à d’autres facteurs pronostiques spécifiques.

**Méthodes:**

Un profil immunohistochimique de 32 TNPs fixées au formol et incluses en paraffine, diagnostiquées entre Janvier 2007 et Décembre 2010, a été réalisé.

**Résultats:**

Nos résultats ont mis en évidence l’association des TNPs n’exprimant pas le CD44s avec une perte de différenciation et une progression tumorale et nous avons rapporté une association significative entre l’absence d’expression du CD44s et la présence de métastases. Nous avons également constaté que l’expression du CD44s définit des sous-groupes de patients dans les tumeurs n’amplifiant pas le MYCN, comme en témoigne son association avec les stades INSS bas, l’absence de métastases et l’histologie favorable de Shimada.

**Discussion:**

Ces résultats appuient l’hypothèse du rôle de la glycoprotéine CD44s dans le potentiel de croissance invasive des cellules néoplasiques et suggèrent que son expression pourrait être prise en considération dans des voies thérapeutiques ciblant les métastases.

## Introduction

Peripheral neuroblastic tumors (pNTs) are a generic term including neuroblastoma (NB), ganglioneuroblastoma (GNB), and ganglioneuroma (GN). These embryonic tumors, which account for 15% of childhood cancer fatalities, are derived from immature sympathetic neuroblasts and diagnosed in the primary sites related to the distribution of neural crest cells, such as adrenal medulla and structures of the sympathetic nervous system in the thorax, abdomen, and pelvic cavity [1].

The most benign tumor is the ganglioneuroma, which is composed of gangliocytes and mature Schwannian stroma. Ganglioneuroblastoma is composed of both mature gangliocytes and immature neuroblasts and has intermediate malignant potential. Neuroblastoma is the most immature, undifferentiated, and malignant tumor of the three [2].

These tumors are well known to show unique and often unpredictable clinical behaviors. Recent advances have led to the belief that the pNTs are heterogeneous and contain biologically different tumors. The biologically favorable tumors have a potential of involution/spontaneous regression or tumor maturation with or without chemotherapy/irradiation therapy. In contrast, the biologically unfavorable tumors progress aggressively and often bring a fatal outcome to the patients despite intensive treatment [3].

The International Neuroblastoma Pathology Classification (INPC), based on the Shimada classification system adopted in 1999 [4,5] and revised on 2003 [6], distinguishes favorable and unfavorable tumors depending on the age at diagnosis, the amount of Schwannian stromal content, the degree of neuroblastic differentiation and the mitosis- karyorrhexis index (MKI) [7].

In addition to the modified Shimada classification [8], other specific markers, alone or in combination, can help to stratify disease by adding prognostic significance to pNTs. At the moment, stratification is based on clinical features: age at diagnosis (children older than 18 months have a better prognosis compared to infants) [9] and International Neuroblastoma Staging System (INSS) classification (patients with localized tumors have an excellent outcome) [10]. Genetic features are also involved in these tumors, the most independent prognostic factor being the MYCN proto-oncogene status: hence, MYCN amplification (≥10 copies) is highly correlated to very aggressive disease [11] whereas copy number gain (1–9 copies more than chromosome 2 centromer signals) is recently reported to correlate with good outcome; indeed, Zhou et al. demonstrated in a very recent study (2013) that patients with MYCN gain tumors had a better prognosis than those presenting MYCN amplification and even those with MYCN gene normal copy number) [12]. Other biological characteristics having a prognostic value such as TrkA expression (TrkA is expressed at high levels in biologically favorable neuroblastomas) [13], ploidy (in some groups of children with NB, near-triploid DNA content tumors are prognostically favorable in contrast to tumors with a near-diploid DNA content) [14] and chromosomal abnormalities (tumors harboring segmental chromosome alterations (1p and 11q losses or 17q gain) are considerably worse than those with only numerical chromosome aberrations (whole chromosome gains or losses) [15-17], have also been detailed in pNTs tumors.

CD44 is an adhesion molecule with binding domains for hyaluronic acid (HA), an abundant and crucial component of the extracellular matrix, and other glycosaminoglycans such as collagen, laminin and fibronectin [18,19]. CD44 comprises a family of glycoproteins that are encoded by 20 exons of a single gene located at the short arm of chromosome 11 [20]. The size of the molecule varies between 80 and 200 kDa. This size heterogeneity is due to the variable N- and O-linked glycosylation and to alternative splicing that allows the insertion of the so-called variable exon products (CD44 v) in the extracellular, membrane proximal region of the molecule [20]. The smallest CD44 molecule, which lacks the entire variable region, is standard CD44 (CD44s). As it is expressed mainly on cells of lymphohematopoietic origin, CD44s is also known as hematopoietic CD44 (CD44H). This standard isoform is present on the membrane of most vertebrate cells [18]. In contrast, expression of isoforms including some variants, characterizes rapid renewal tissues such as skin [20]. CD44 cell surface transmembrane glycoprotein has been involved in many cellular processes which include lymphocyte activation, recirculation and homing, adhesion of extracellular matrix, angiogenesis, cell proliferation, cell differentiation and cell migration [21,22]. CD44 is highly expressed in many malignancies and is correlated with the tumor biological behavior including tumorigenesis, growth, metastasis and prognosis [23]. It is a reliable indicator of tumor load and disease activity; it is also called metastasis associated protein. Many correlative studies have revealed pronounced expression of CD44 in human tumors, and in some, such as colon cancer, clear cell carcinoma of the kidney or hematological malignancies, the expression of some variants has a poor prognosis [24-26]. Conversely, the degree of malignancy of some cancers such as prostate cancer, is associated with loss of CD44 [27], suggesting that CD44 could be a tumor suppressor. CD44 is, therefore, an attractive biologic marker for analysis in tumors. In addition, the evaluation of its expression by immunohistochemistry is a simple, fast and reliable standardized method. The aim of this study was to assess retrospectively the clinical relevance of CD44s cell-surface expression in a series of human peripheral neuroblastic tumors and to correlate this expression with other clinical, histological and biological features characterizing these pNTs. There was also an attempt to report the current data concerning this cancer-initiating cell.

## Materials and methods

This retrospective study was performed on 32 formalin-fixed paraffin-embedded pNTs (neuroblastoma, ganglioneuroblastoma and ganglioneuroma) archival material collected from the Pathology Department of the “Ibn Rochd” Hospital. Tissue specimens corresponded to cases, diagnosed between January 2007 and December 2010, with clinical data was retrieved from the files of the Department of Pediatric Oncology and Hematology of the “20 Août 1953” Hospital and from the Departments of Pediatric Visceral Surgery and Pediatrics III of the “Harouchi” Children’s Hospital. These three hospitals form the “Ibn Rochd” University Medical Center of Casablanca, Morocco.

Patients were classified according to the INSS classification that classified pNTs into five stages in terms of the clinical presentation and age. Briefly, stages I and II are localized tumors, stage II with ipsilateral lymph node involvement. Stage III tumors infiltrate across the midline or show contralateral lymph node involvement. Stage IV is metastatic disease. Stage IVs (“s” for special), corresponding to localized tumors with a special pattern of liver, skin and/or minimal bone marrow metastasis (without bone involvement), is restricted to infants (under 1 year). Stages I, II and IVs are considered favorable, whereas stages III and IV are associated with poor prognosis.

## Histopathological review

After the morphological examination of the hematoxylin and eosin (H&E) stained slides of the untreated pNTs, the tumors were classified in accordance with the INPC (Shimada system). This system classifies the tumors based on the amount of Schwannian stroma into three categories: neuroblastomas are stroma-poor; ganglioneuroblastomas are stroma-rich (GNB intermixed) or stroma-poor and stroma-rich/dominant (GNB nodular) and ganglioneuromas are stroma-dominant.

Based on the proportion of differentiating neuroblasts (the degree of neuroblastic maturation toward ganglion cells) and the presence or absence of neuropil (thin neuritic processes), the neuroblastomas can be subdivided into three subtypes. Undifferentiated neuroblastomas show no signs of maturation; they contain less than 5% differentiated neuroblasts and no neuropil. The poorly differentiated neuroblastomas also contain less than 5% differentiating neuroblasts; they differ from the undifferentiated tumours by the distinct presence of neuropil among the tumor cells. Schwannian stroma-poor tumors containing more than 5% differentiating neuroblasts are classified as differentiating neuroblastomas.

The ganglioneuroblastomas are divided into two subtypes: Ganglioneuroblastoma intermixed is a stroma-rich tumour with microscopic well defined nests of neuroblasts in different stages of maturation as well as varying numbers of maturing ganglion cells found in the background of neuropil. Ganglioneuroblastoma nodular has a macroscopically visible well-demarcated nodule of stroma-poor neuroblastic tissue in an otherwise stroma-rich or stroma-dominant tissue composed of maturing ganglion cells. Ganglioneuroma mature is the completely mature lesion, containing only mature ganglion cells and Schwannian stroma.

Three MKI classes (low: 2% or 100 of 5000 mitotic and karyorrhectic cells; intermediate: 2-4 % or 100–200 of 5000 mitotic and karyorrhectic cells and high: 4% or 200 per 5000 mitotic and karyorrhectic cells) were distinguished. According to Shimada classification, pNTs were placed either in the favourable histology (FH) or the unfavourable histology (UH) category. Table 1 summarizes the classification according to Shimada.

**Table 1:** The distribution of tumor subtypes

**Subtypes**	**n = 32**
Undifferentiated neuroblastoma	7
Poorly differentiated neuroblastoma	9
Differentiating neuroblastoma	2
Ganglioneuroblastoma intermixed	5
Ganglioneuroblastoma nodular	7
Ganglioneuroma	2

## CD44s expression by immunohistochemistry

Briefly, 4 μm thick sections were cut from the formalin-fixed, paraffin-embedded specimens and placed on treated slides. After having been heated at 60°C for 30 min, then at 37°C overnight, the tissue sections were dewaxed in toluene for 10 min, three times each, rehydrated in ethanol for 10 min, three times each, and rinsed once in distilled water for 5 min. Antigen availability for CD44 was enhanced by pretreatment for 30 minutes at 99°C in citrate buffer (pH 6). After 20 minutes cooling at room temperature, endogenous peroxidase was quenched with H_2_O_2_ for 10 min. Sections were then incubated with monoclonal primary antibody for 30 min. Primary antibody used in this study was anti-CD44-standard form (1:50 dilution) (clone DF1485, Dako). Immunobinding of anti-CD44 was visualized with Dako REAL EnVision Detection System, Peroxidase/DAB, Rabbit/Mouse (DAKO kit, Dako, Glostrup, Denmark). Sections were counterstained with Hematoxylin for 1–2 min and mounted with Eukitt.

Multiple Myeloma with known CD44s positivity served as positive control.

## Immunostaining assessment of CD44s expression

All of immunostained tissues were scored semi-quantitatively by one author (N.T.J.) without the knowledge of the clinical and pathologic parameters of patients. Obtained results were confirmed by two observers (N.T.J. and S.Z.) on two separate occasions. In difficult cases, a consensus was achieved using a multi-headed microscope.

Slides were first observed at 10 × magnification to obtain an impression of the overall distribution of the neoplastic cells. Positive staining was then assessed semi-quantitatively at higher magnifications. CD44 expression was considered positive if brown membrane staining was found in more than 10% of neoplastic cells. In almost all positive tumor samples, staining was strong and uniform.

## MYCN amplification status by fluorescence *in situ* hybridization (FISH)

FISH was performed on formalin-fixed, paraffin-embedded tissue. The sample that contained the most immature areas was selected, particularly in GNB, which displayed heterogeneous maturation. FISH was performed using a commercial Locus Specific Identifier (LSI) MYCN SpectrumGreen DNA probe and an alpha satellite region chromosome 2-specific (CEP 2), SpectrumOrange DNA probe as an internal standard. Sections 4 μm-thick were mounted on SuperFrost slides. The sections were dewaxed in three washes of toluene, followed by three washes in ethanol for 10 min at room temperature. Slides were then incubated for 15 min at 99°C in heat pretreatment solution, pH7 (Zymed Laboratories, San Francisco, CA, USA), and digested with enzyme reagent (Zymed Laboratories) at 37°C for 15 min. Each step was followed by two or three washes with phosphate buffered saline (PBS) 10X. After dehydration in a series of 70%, 90% and 100% ethanol for 2 minutes each at room temperature, 10 μl of probe mixture which contained 1 μl of MYCN probe (LSI N-MYC/CEP2, Vysis Abbott), 2 μl of purified H_2_O, and 7 μl of LSI hybridization buffer (Vysis, Abbott), were applied to each target area and slides were glass coverslipped and sealed with RubberCement. Co-denaturation and hybridization were proceeded with the HYBrite (Dako) already programmed (co-denaturation for 5 min at 95°C and hybridization overnight at 37°C). After removing the coverslips, the slides were washed in 2X standard saline citrate (SSC)/0.3% Igepal (Sigma-Aldrich, St. Louis, MO, USA) at 75°C for 5 min and immersed twice in the same solution at room temperature. After air drying slides in darkness, the hybridization was visualized by applying 12 μl of DAPI/Vectashield (Vector Laboratories) mounting medium. The amplification of MYCN was assessed by the number of fluorescent hybridization signals within the nuclei of tumor cells. Tumor cells with more than 10 copies of MYCN were considered as amplified.

## Statistical analysis

Statistical comparisons between subgroups were made using the appropriate statistical test (Chi-square, Chi-square with Yates correction or Chi square for linear trend tests and Fisher exact test). For multivariate analysis, we used the logistic regression with stepwise likelihood ratio test: (Forward: LR (Likelihood Ratio) Method of covariate entry). The analysis was performed by SPSS 16.0 software. For all tests, *P* values lower than 0.05 were considered statistically significant.

## Results

### Clinical presentation

Thirty-two tumor specimens from newly diagnosed peripheral neuroblastic tumors patients were collected since 2007 during the 4-year period. The youngest patient was 9 days old, the oldest patient was 13 years old (mean age at diagnosis 41 months). Most analyzed cases, 21/32 (65.6%), were diagnosed among children above 18 months of age. There were 22 (69%) boys and 10 girls (31%), M:F = 2.2. Primary site of the tumor was not detected for one patient. 28.1% of the patients had low stage disease (6 *INSS* stages I/II and 3 *INSS* stage IV-s) whereas 71.9% had high stage (5 *INSS* stage III and 18 *INSS* stage IV). In 71% of cases, tumors were situated in the retroperitoneal space.

## Histopathology

All 32 pNTs were initially classified into four categories: neuroblastoma (NB; Schwannian stroma-poor); ganglioneuroblastoma, intermixed (GNBi; Schwannian stroma-rich); ganglioneuroma (GN; Schwannian stroma-dominant); and ganglioneuroblastoma, nodular (GNBn; composite, Schwannian stroma-rich/stroma-dominant and stroma-poor). The distribution of tumor subtypes is shown in Table 1.

## Shimada prognostic grading

19 (59.4%) tumors showed unfavourable histology whereas 13 (40.6%) were classified into favorable histology. The nodular ganglioneuroblastoma had neuroblastic nodules with unfavorable histology and was included in this category. 88.9% tumors with FH were localized (stage I, II or IV-s). FH was associated with low clinical stage (*P* = 0.002). The opposite was true for the tumors with UH, with only 11.1% localized while 78.3% widespread at diagnosis. UH was associated with high clinical stages (stage III or IV) (Table 2).

**Table 2:** Prognostic types of neuroblastic tumors based on the INPC in relation to INSS

	**Favorable histology**	**Unfavorable histology**	***P *value**
**Low stages**	8 (88.9)	1 (11.1)	0.002*
**(I, II, IV-s)**			
**High stages**	5 (21.7)	18 (78.3)	
**(III, IV)**			

* Yates correction for continuity.

## Estimation of prognostic value of CD44 cell-surface expression in correlation with prognostic factors and histoclinical features in neuroblastic group of tumors

**Figure 1:**
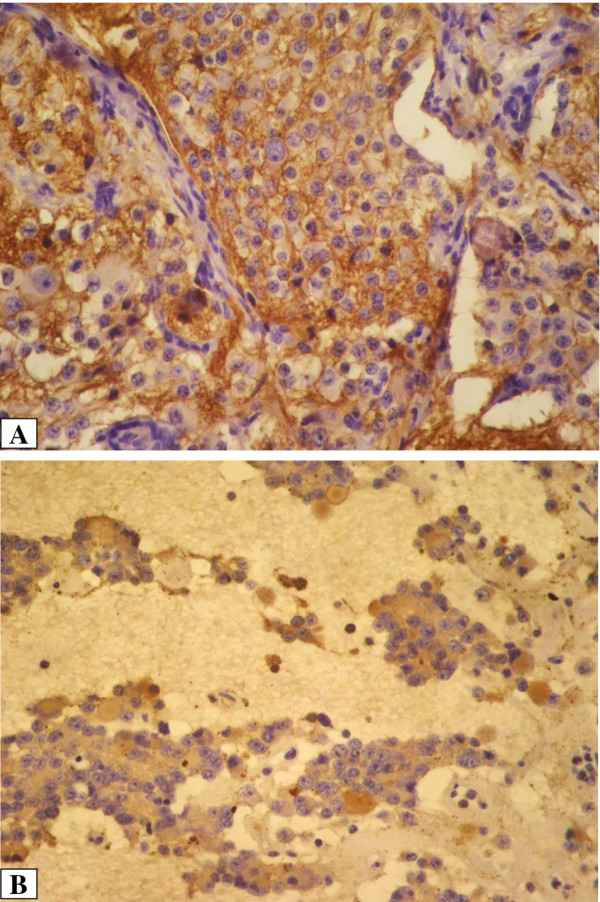
**CD44s expression on neuroblastoma cells: A/membranous staining. B**/cytoplasmic staining (magnification x400).

Positive CD44 immunostaining was observed on 18 samples (56%) (Figure 1). Essential correlations between CD44 expression and histoclinical characteristics in the neuroblastoma group of tumors are shown in Table 3.

**Table 3:** Essential correlations between CD44 cell-surface glycoprotein expression and histoclinical features

**Characteristics**	**Specimens No (%)**	**Immunohistochimical expression of CD44 protein**		***P value***
		**Negative (%)**	**Positive (%)**	
**Age (months)**				
< 18	11 (34.4)	5 (45.5)	6 (54.5)	1*
≥ 18	21 (65.6)	9 (42.9)	12 (57.1)	
**Primary site**				
Retroperitoneal space	22 (71)	9 (40.9)	13 (59.1)	1*
Other sites	9 (29)	4 (44.4)	5 (55.6)	
**Metastasis**				
Presence	21 (65.6)	13 (61.9)	8 (38.1)	0.013*
Absence	11 (34.4)	1 (9.1)	10 (90.9)	
**INSS stages**				
I, II, IVs	9 (28.1)	0 (0.0)	9 (100.0)	**0.006**
III, IV	23 (71.9)	14 (60.9)	9 (39,1)	
**Differentiation**				
NB	18 (56.2)	11 (61.1)	7 (38.9)	**0.025**
GNB + GN	14 (43.8)	3 (21.4)	11 (78.6)	
**Shimada category**				
FH	13 (40,6)	1 (7.7)	12 (92.3)	**0.001**
UH	19 (59,4)	13 (68.4)	6 (31.6)	

* Yates correction for continuity.

No correlations between age at diagnosis or primary sites of the disease and CD44 expression were observed (P >0.05). All the low grade stages (I, II and IVs) were CD44-positive. The significant correlation of CD44 expression with the stage of the disease strongly suggests that it might help predict clinical outcome (*P* = 0.006).

Of the 32 tumors, 18 were undifferentiated, whereas 14 were classified as differentiated pNTs (ganglioneuroblastoma or ganglioneuroma). CD44 expression was observed on 78.6% differentiated tumors, but only on 38.9% of undifferentiated specimens (*P* = 0.025). In contrast, only 21.4% differentiated tumors showed no CD44-staining versus 61.1% undifferentiated cases. We also observed a significantly statistical correlation between CD44 expression and Shimada’s histological grading (*P* = 0.001): CD44s negative tumors are associated with an unfavorable prognostic type.

The CD44 reactivity on tumor samples was significantly correlated with the absence or presence of metastasis: among cases with no metastases, 90.9% expressed CD44 against only 9.1% of cases CD44-negative (*P* = 0.013).

The expression of investigated CD44 protein was compared with the above described selected histoclinical and prognostic factors. The logistic regression model has allowed identifying two variables associated with the expression of CD44, Shimada category and the presence of metastases (Table 4). The patients without metastasis and those of the FH group were more likely to express CD44.

**Table 4:** Results of multivariate analysis of CD44 expression compared with histoclinical features

	***OR***_***a***_	***P value***
**Metastasis**	0.07	**0.037**
**Shimada category**	23.16	**0.011**

*OR*_*a*_*:* Adjusted odds ratio.

## CD44 expression and MYCN amplification status

Analysis of CD44 expression and MYCN status was available for 31 children. Of 18 CD44-positive patients, 15 (83.3%) showed no MYCN amplification and only 3 (16.7%) were MYCN-amplified. In contrast, of 13 CD-44 negative patients, 6 (46.1%) showed no MYCN amplification and 7 (53.9%) were amplified. However, these differences were not significant (*P* = 0.073). Table 5 shows the results of the CD44 expression stratified by the MYCN amplification status in relation to clinical and histopathological parameters. We noted that when MYCN was non-amplified, CD44 expression was significantly associated with low INSS stage (*P* = 0.046), absence of metastasis (*P* = 0.019) and favorable INPC histology (*P* = 0.004). In contrast, in MYCN-amplified tumors, no significant association between CD44 expression and the other factors had been demonstrated.

**Table 5:** CD44 expression stratified by the MYCN amplification status in relation to other histoclinical parameters of pNTs

	**MYCN amplified**		***P value***	**MYCN nonamplified**		***P value***
	**CD44 positive**	**CD44 negative**		**CD44 positive**	**CD44 negative**	
	**No. (%)**	**No. (%)**		**No. (%)**	**No. (%)**	
**Age (months)**						
< 18	1 (20)	4 (80)	1**	4 (80)	1 (20)	1**
≥ 18	2 (40)	3 (60)		10 (66.7)	5 (33,3)	
**INSS stages**						
I, II, IVs	1 (100)	0 (0)	0,3	8 (100)	0 (0)	**0,046**
III, IV	2 (22.2)	7 (77,8)		7 (53.8)	6 (46,2)	
**Metastasis**						
Presence	2 (25)	6 (75)	1	6 (50)	6 (50)	**0,019**
Absence	1 (50)	1 (50)		9 (100)	0 (0)	
**Differentiation level**						
Undifferentiated	3 (30)	7 (70)	-	5 (55.6)	4 (44.4)	0,33
Differentiated	0 (0)	0 (0)		10 (83.3)	2 (16.7)	
**Shimada category**						
Favorable histology	1 (50)	1 (50)	1	11 (100)	0 (0)	**0.004**
Unfavorable histology	2 (25)	6 (75)		4 (40)	6 (60)	
**MKI Index**						
High	1 (50)	1 (50)	0.645*	5 (71,4)	2 (28.6)	0.262*
Intermediate	0 (0)	2 (100)		2 (50)	2 (50)	
Low	1 (25)	3 (75)		1 (33.3)	2 (66.7)	

* chi square for linear trend.

** Fisher exact test.

## Discussion

In this first histopathological review of Morocco, we have investigated the prognostic value of CD44 cell-surface glycoprotein in 32 peripheral neuroblastic tumors and its relationship to other known prognostic indicators such as age at diagnosis, *INSS* stage of the disease, primary site, metastasis, differentiation and modified Shimada system.

An earlier study by Combaret et al. showed that CD44 expression strongly correlated with patient’s age as it was expressed on 61 of 66 tumors from infants (less than 1 year of age), but only in 47 of 74 tumors from older children (P < 0.0001) [28]. We were unable to demonstrate such a correlation probably because of the high mean age of our study cohort (41 months) while the frequent median age of pNTs diagnosis is 22 months [29]. This disparity may be due to a delay in diagnosis caused by difficulties in access to health care. Moreover, the study of Combaret et al., achieved in 1997, had investigated the CD44 expression compared to the 1 year cut-off, while a recent study showed evidence for an age cut-off (between 15 and 19 months) greater than 1 year for use in risk stratification of neuroblastoma patients [9]. So we definitely retained the threshold of 18 months in our study.

Study achieved by Taran et al. did not find a clear statistically significant correlation between CD44 expression and histoclinical parameters and currently known prognostic factors; however, this study had reported CD44 expression in 88.88% of cases and, unlike our study, it has noticed that the strongest CD44 expression was observed in tumors situated in the retroperitoneal space [30].

As reported by several other studies [31,32], we found that CD44 is down-regulated in advanced neuroblastomas (stage III and IV), whereas the earlier and prognostically favorable stages (I, II, IVs) are characterized by tumor cells maintaining their ability to synthesize the standard form of CD44 (*P* = 0.006). This significant correlation of CD44 expression with the *INSS* stage strongly suggests that it might help in the prediction of clinical outcome. In contrast, another study demonstrated no major variation of CD44 incidence throughout the stages, except for stage IVs (I-II-III: 84%, IVs: l00%, IV: 85%) [33]. We can then speculate that CD44 might positively contribute to maturation and spontaneous regression of low grade stages I, II and IVs neuroblastic tumors.

In our study, absence of CD44s expression correlated significantly with a lack of differentiation (*P* = 0,0025). This result was comparable with other studies on neuroblastic tumors, indicating that the expression of CD44 standard form is linked to tumor maturation and differentiation [34,35]. These findings were supported by a study which reported that cultured human neuroblastoma GOTO cells, of which a cell adhesion molecule CD44 expression is usually suppressed, could be induced to differentiate into Schwannian cells and neuronal cells in the presence of 5-bromo-2’-deoxyuridine (BrdU) and by serum depletion respectively. These GOTO cells differentiated into Schwannian cells, specifically expressed CD44 glycoprotein, while this molecule remained suppressed in cells differentiated into neuronal cells which suggests that CD44 might play an important role in GOTO cells differentiation into Schwannian cells [36] and thus in tumor evolution into maturing subtypes.

Our data analyzing the expression of CD44s in 32 neuroblastic tumours according to the INPC system (modified Shimada grading), demonstrated that 92.3% of CD44s-positive tumors were of a favorable prognostic type based on the INPC (with a better event-free survival probability) versus 68.4% CD44s-negative tumors in the unfavorable prognostic category (poorer event-free survival probability) (*P* = 0,001). These data were in agreement with another study in which approximately 89.5% of CD44s- negative pNTs were associated with the unfavorable prognostic INPC system in comparison with CD44s-positive tumors (approximately 65.5%) (*P* <0.05) [37]. Several other groups have shown the same correlation [32,35]. Therefore, absence of expression of CD44s can be seen in the more immature peripheral neuroblastic tumors, which correspond with the worst prognostic type based on the modified Shimada system [6].

Cell adhesion is the condition *sine qua non* for the development of multicellular organisms. Cell migration, under physiological conditions, is the most important during embryogenesis, in tissue remodelling, wound healing and leukocyte migration [38]. Cell adhesion and migration are critical steps in cancer progression.

Simplistically, metastasis is depicted as the sequential dissociation of tumor cells from the primary tumor, migration of the dissociated cells into and through normal tissue, intravasation, survival in circulation, extravasation, migration into and through extracellular matrices, tumor cell proliferation, or, for tumor cells with high invasive potential, proliferation, invasion, and uncontrolled progression at this and/or multiple other sites [38,39]. In neuroblastic tumors, metastases are found mainly in the bone marrow, bone and lymph nodes [3], however, other less frequent secondary sites such as the liver, kidneys, lungs and even the heart have been reported [40]. Many cell adhesion molecules including integrins, cadherins, immunoglobulin (Ig)-like CAMs, selectins, miscellaneous others and CD44, have been reported to be involved in each of these steps of the metastatic process and to be expressed by primary neuroblastoma cells or cell lines and associated with a given function or phenotype in this cell type [39].

The role of CD44 adhesion glycoprotein in the evolution and progression of cancer has received a lot of attention in recent years [38,41]. In view of the fact that hyaluronic acid is the major component of the extracellular matrix and CD44 is the major receptor for HA [19], it is not surprising that CD44 plays a main role in HA adhesion and crawling along the HA matrix thereby providing a mechanism by which CD44 could influence adhesion and de-adhesion to the extracellular matrix [21]. It is believed that elevated HA levels form a less dense matrix and consequently enhance cell motility as well as invasive ability into other tissues [19]. The HA–CD44 interactions have a central role in receptor tyrosine kinase (RTK)-induced activation of anti-apoptotic pathways and actively promote tumor cells and possibly cancer-initiating cell survival through their associations with multidrug resistance genes [40]. Importantly, activation of signalling pathways initiated by the tumor matrix could be inhibited by HA degradation, by competition with small HA fragments, by CD44 blockade or by CD44 knockdown [41].

In most cancers, the dysregulated expression of CD44 is not the result of CD44 mutations. Instead, genes that are implicated in promoting carcinogenesis control the patterns of CD44 expression in cancer cells. Alternative splicing, for example, is under the control of mitogenic signals including the Ras-MAP kinase cascade. In addition, the loss of different subunits of the SWI/SNF chromatin remodeling complex, which are mutated in numerous cancers, results in the loss of CD44 transcription [21]. Aberrant CD44 expression is therefore inextricably linked to genetic alterations that lead to tumor growth and metastasis.

High expression of CD44 was observed on the surface of skin, cervix, endometrium, stomach, colon and prostate cancer cells [22,30]. It has been described in the literature that the presence of the CD44 molecule allows neoplastic cells to metastasize. However, results from other research have proved that it is not an increase but a decrease of CD44 expression that appears as an unfavorable prognostic factor in bladder cancer [42,43].

Our data reported a statistical significant association (*P* = 0.013), between CD44 positive expression and the absence of metastasis, on human neuroblastic cells. A study by Valentiner et al. reported that expression of CD44 was associated with a metastatic pattern of the neuroblastoma cell lines engrafted in the SCID mice and that CD44-negative neuroblastomas developed numerous micrometastases in the lung interstitium while CD44-positive neuroblastomas produced multicellular metastases predominantly located in the intra- and periarterial space of the lung [44]. These data have been contradicted by a more recent report showing that the CD44 negative SK-N-SH neuroblastoma cells were the ones that infiltrated the bone marrow, spleen and liver of transplanted animals, clearly indicating that the metastatic ability of neuroblastoma cells is independent of CD44 [45]. This study also showed that lack of CD44 expression was accompanied by lower levels of various adhesion molecules, including CD49d (α4 integrin), CD49e (α5 integrin) and CD29 (β1 integrin), as well as ICAM1 and neural cell adhesion molecule NCAM [45] which reduce adherence capacity and may enhance the cells migratory ability and their propensity to form metastases [46]. Therefore, the distinct cell adhesion profile of CD44s negative cells suggests an enhanced metastatic potential.

## Conclusions

In conclusion, and as confirmed by multivariate analysis, overexpression of CD44s could be regarded as a powerful predictor of the absence of tumor infiltration and of the prognostically favorable subtypes of neuroblastic tumors. Our results validate the prognostic value of CD44s expression not only in neuroblastoma, as reported in some previous works, but in all human neuroblastic tumors and we recommend its integration to predict metastasis and to target pathways for potential therapeutic strategy.

## Competing interests

The authors declare that they have no competing interests.

## Authors’ contributions

NTJ and IT participated in the design of the study, carried out the immunohistochemical analyzes and drafted the manuscript. HS, MI, SB and SZ acquired clinical data. ZS and MBO performed the statistical analysis. All authors approved the final manuscript.
